# Integrated haemodynamic monitoring of fluid responsiveness and fluid tolerance in critical care: from physiology to bedside applications

**DOI:** 10.1007/s10877-026-01467-3

**Published:** 2026-07-10

**Authors:** Henrique Mendes, Marc Zentar

**Affiliations:** 1 School of Clinical Medicine, University of New South Wales, Sydney, NSW Australia; 2 Department of Intensive Care Medicine, St George Hospital, Sydney, NSW Australia

**Keywords:** Fluid responsiveness, Fluid tolerance, Haemodynamic monitoring, Monitoring platforms, Venous excess ultrasound, Point-of-care ultrasound

## Abstract

The framework of fluid responsiveness addresses only whether volume expansion will augment cardiac output, not whether the circulation can accept that volume without producing congestive organ injury. The complementary concept of fluid tolerance has accumulated empirical support over recent years and is integral to understanding patients’ fluid status. This narrative review synthesises the physiological basis of fluid responsiveness and fluid tolerance, describes the bedside monitoring tools available to assess each, and considers the clinical implications of their joint interpretation in critical care. We performed a literature search restricted to articles published between 2020 and the date of the search. The retrieved abstracts were evaluated for relevance, and their reference lists were subsequently screened to identify additional pertinent publications. Fluid responsiveness and fluid tolerance are dissociable but physiologically interdependent, sharing determinants such as biventricular function, stressed volume and venous capacitance. Dynamic tests remain the preferred approach for assessing preload responsiveness; each has specific applicability conditions, and many require continuous or rapidly repeated cardiac output monitoring. Emerging tools have been developed to assess fluid tolerance, primarily based on vascular sonography; their interpretation requires concurrent assessment of biventricular function and further validation. Reframing fluid stewardship around the interaction between responsiveness and tolerance shifts the focus from forward-flow alone to net physiological benefit. No single parameter or device fully captures fluid status. An integrated, serial, multi-parametric assessment supported by appropriate monitoring platforms is therefore preferable to reliance on any single measure. At present, these tools support fluid stewardship rather than representing interventions with independently proven survival benefit. Future priorities include defining organ-specific congestion thresholds and testing whether monitoring-informed protocols improve outcomes.

## Introduction

Fluid resuscitation is a first-line intervention in circulatory shock, aimed at restoring tissue perfusion by augmenting cardiac output and oxygen delivery [1]. Over three decades of investigation, the concept of fluid responsiveness has come to dominate bedside decision-making, providing a physiological framework to identify whether volume expansion will meaningfully increase stroke volume [2]. This paradigm focuses predominantly on forward flow and largely neglects the venous circulation, where fluid administration may produce congestion, interstitial oedema and organ dysfunction. The clinical stakes are substantial: in critically ill adults, positive cumulative fluid balance and fluid overload are associated with adverse outcomes, including fewer ventilator-free days, acute kidney injury and increased mortality [3–5]. Conversely, inadequate early haemodynamic support may perpetuate tissue hypoperfusion and worsen organ dysfunction [1].

The complementary concept of fluid tolerance, more recently formalised [6], shifts attention from whether the heart can accept more volume to whether the circulation and organs can accommodate it without harm. Fluid therapy increases both forward flow and back pressure, and the net clinical benefit of any given intervention depends on which effect predominates. Nevertheless, responsiveness and congestion do not lie on a single continuum: recent data indicate that venous congestion and fluid responsiveness frequently coexist in critically ill patients [7, 8].

Fluid stewardship has been defined as a coordinated approach to optimising the selection, dose, rate and duration of intravenous fluid therapy to improve clinical outcomes and minimise harm [9]. In the present review, we use the term more narrowly to describe serial, monitoring-informed fluid decisions that balance the potential haemodynamic benefit of volume expansion against the risk of congestive organ injury.

Recent narrative reviews have addressed individual monitoring domains, including central venous pressure (CVP) interpretation [10], functional haemodynamic tests for fluid responsiveness in critically ill patients [11], and the foundational physiological principles of perioperative and intensive care haemodynamic monitoring [12]. Despite the physiological interdependence between responsiveness and tolerance, there is scarce literature on their integrated monitoring. This review describes the physiological basis of fluid responsiveness and fluid tolerance and their complementary roles in determining fluid status. It then maps the bedside monitoring tools available to assess each (Table 1), analyses their shared determinants, and argues that integrating flow and congestion signals is central to bedside fluid stewardship.

## Methods

We searched MEDLINE (via PubMed), Embase, and Google Scholar, restricting our primary query to English-language studies of adults published between 1 January 2020 and 30 March 2026. The search used free-text keywords and related synonyms for “fluid tolerance”, “fluid responsiveness”, and “critical care”, combined with appropriate Boolean operators. The initial search yielded 853 results. After removing duplicates and screening 548 abstracts, we selected 72 articles for full-text appraisal based on relevance to physiological mechanisms, monitoring technology, bedside applicability, and association with clinical outcomes. We supplemented this with seminal older literature and directly relevant studies identified through citation tracing and peer review.

## Results

### Fluid responsiveness: physiological basis

Fluid responsiveness is defined as the cardiovascular system’s ability to increase cardiac output (typically by 10 to 15%) in response to an increase in preload [2]. Its physiological basis lies in the Frank–Starling mechanism: when a ventricle operates on the steep, ascending portion of its function curve, an increase in end-diastolic volume produces a proportional rise in stroke volume through length-dependent activation of the myofilaments, whereby increased sarcomere length enhances calcium sensitivity of the contractile apparatus [13]. Once the ventricle reaches a plateau, further volume loading fails to augment output and instead raises filling pressures, worsening venous congestion without haemodynamic benefit [14]. Assessment of fluid responsiveness, therefore, seeks to determine, before any fluid is administered, where on this curve the patient’s ventricle is operating.

The cardiovascular system functions as a closed loop in which venous return equals cardiac output at steady state. The upstream driving pressure for venous return is mean systemic filling pressure (Pmsf): the equilibrium pressure that would exist throughout the systemic circulation if flow were to stop. Pmsf is determined by stressed blood volume, the fraction of intravascular volume generating pressure against vessel walls, and by systemic vascular compliance; unstressed volume fills venous capacitance without contributing to Pmsf [15]. The downstream opposing pressure is right atrial pressure (RAP). Thus, the gradient between Pmsf and RAP, divided by the resistance to venous return, determines venous return and therefore cardiac output at steady state [16].

Fluid administration may increase Pmsf by increasing stressed volume, whereas vasopressors may recruit unstressed volume through venoconstriction while also altering arterial tone and ventricular afterload. Their net haemodynamic effect depends on how these interventions modify the relationship between upstream pressure, RAP, cardiac function and organ perfusion [17]. Effective preload increases only if volume expansion raises Pmsf more than RAP. If RAP rises by a similar or greater amount, the gradient for venous return does not increase, the ventricle receives no additional distending pressure, and stroke volume may not rise [15]. Thus, fluid responsiveness reflects not only ventricular preload reserve but also the interaction between cardiac function and venous return. Heart rate further modulates this relationship through its effects on diastolic filling time, ventricular filling and time-averaged venous return.

### Bedside tools for assessing fluid responsiveness

Static markers of preload, including CVP and pulmonary artery occlusion pressure, have repeatedly been shown to be poor predictors of the haemodynamic response to volume expansion [18, 19]. This limitation has driven the development of dynamic tests, which exploit transient, reversible changes in preload to probe the slope of the Frank–Starling curve without committing to a full fluid bolus [11, 20].

Heart–lung interaction indices exploit the cyclical effects of positive-pressure ventilation on intrathoracic pressures and cardiac loading. During mechanical insufflation, increased intrathoracic pressure transiently impedes venous return, reducing right ventricular preload; this translates, after a few heartbeats, into a fall in left ventricular stroke volume. In preload-responsive patients, these respiratory-induced oscillations in stroke volume are amplified, producing measurable swings in arterial pulse pressure (pulse pressure variation, PPV) and stroke volume (stroke volume variation, SVV). A PPV of 12 to 15% is associated with a subsequent increase in cardiac output after volume expansion [21], but this threshold requires controlled conditions, including tidal volumes of at least 8 ml.kg⁻¹ of predicted body weight, absence of spontaneous respiratory effort and preserved sinus rhythm [22]. In contemporary critical care, where lung-protective ventilation, spontaneous triggering and atrial fibrillation are common, a substantial proportion of ventilated patients falls outside the reliable operating range of PPV at any given time [22, 23]. Continuous display of arterial-waveform PPV can support trend-based interpretation when these operating conditions are met, provided grey-zone values are not over-interpreted [22]. Non-invasive finger-cuff systems can also derive beat-to-beat PPV. Evidence for non-invasive finger-cuff PPV is more limited. One post-cardiac-surgery validation cohort found close correspondence with intra-arterial PPV but occasional misclassification [24], whereas a systematic review found that finger-cuff and invasive measurements of arterial pressure and cardiac output are not interchangeable in surgical or critically ill patients [25]. Finger-cuff PPV is therefore better treated as a lower-certainty, context-dependent trend signal than as a basis for threshold-based classification.

Several complementary dynamic tests have been developed to extend the range of patients in whom preload responsiveness can be assessed reliably. The tidal volume challenge, in which tidal volume is transiently increased from 6 to 8 ml.kg⁻¹ and the change in PPV is measured, improves discriminative performance in patients ventilated with low tidal volumes [20, 26]. The end-expiratory occlusion test (EEOT), in which a 15-second occlusion at end-expiration abolishes the cyclic reduction in venous return and increases cardiac output in preload-responsive patients, is less sensitive to tidal volume and rhythm but requires a patient who tolerates a brief apnoea without triggering the ventilator [20]. The mini-fluid challenge, which consists of delivering 100 ml of crystalloid over 1 min with real-time measurement of stroke volume, provides a direct but not reversible test [20, 27].

Passive leg raising (PLR) overcomes many of these limitations by acting as a reversible, endogenous fluid challenge. Elevating the lower limbs from a semi-recumbent position transfers approximately 300 ml of venous blood from the legs and splanchnic circulation into the central compartment, transiently increasing preload [28, 29]. An increase in cardiac output of at least 10% during PLR, measured by a real-time cardiac output monitor, identifies preload responsiveness with high sensitivity and specificity in ventilated and spontaneously breathing patients, and in those with cardiac arrhythmias [30, 31]. The test requires a monitor capable of detecting rapid changes in stroke volume or cardiac output within one to two minutes. The induced change should reverse when the legs are returned to the semi-recumbent position. PLR is contraindicated in patients with raised intracranial pressure or certain lower-limb injuries. End-tidal carbon dioxide has been validated as a surrogate for real-time cardiac output measurement during PLR in mechanically ventilated patients with stable minute ventilation [27]. The choice of dynamic test is therefore conditioned by both patient physiology and the available monitoring platform.

These dynamic tests require a platform capable of detecting rapid changes in stroke volume or cardiac output. Continuous cardiac output monitoring platforms, including arterial waveform analysis (calibrated and uncalibrated) and transpulmonary thermodilution, provide the real-time data needed to interpret dynamic tests and track responsiveness and tolerance over time. The pulmonary artery catheter has evolved from a routine bedside device to one reserved for selected populations such as advanced heart failure, pulmonary hypertension and complex cardiac surgery [32]. It provides thermodilution-derived cardiac output, mixed venous oxygen saturation, and direct measurement of pulmonary artery and occlusion pressures. Across these platforms, available outputs differ substantially in invasiveness, calibration strategy, flow measurement and access to volumetric congestion indices, such as extravascular lung water and global end-diastolic volume. Their outputs are not interchangeable, and the physiological assumptions of each platform must be matched to the clinical question [12, 33]. The choice of platform, therefore, depends on the clinical context, perceived risk of fluid intolerance, and the invasiveness tolerated.

Validation studies have demonstrated that uncalibrated pulse contour devices can detect fluid responsiveness during PLR in critically ill patients, supporting their utility for dynamic test interpretation when calibrated platforms are unavailable, although optimal threshold values for change in cardiac output may differ from those derived for calibrated devices [34]. Because preload responsiveness is itself a transient, decaying state rather than a fixed property, it varies with the stage of resuscitation, fluid infusion rate, vasopressor use, ventilation, and systolic function. Serial and trend-based interpretation is therefore more informative than a single snapshot measurement and may warrant repeated evaluation across the resuscitation window [35]. This longitudinal appraisal holds only when the underlying measurement remains valid for the patient’s physiology and is interpreted alongside other haemodynamic and congestion signals.

### Fluid tolerance: physiological basis

Fluid tolerance can be defined as the capacity to receive fluid without inducing or worsening organ dysfunction [6]. It is not simply a function of volume status but rather of the dynamic interaction among cardiac function, venous capacitance, and downstream organ perfusion. Signals of congestion reflect the cardiovascular system’s inability to accommodate the existing volume at acceptable filling pressures, rather than absolute hypervolaemia per se [36, 37]. Congestion is also heterogeneous: bedside ultrasonographic and haemodynamic signatures can distinguish endotypes with different clinical and outcome profiles [36]. Mechanistically, fluid intolerance arises from four interacting domains.

First, cardiac function determines how a given stressed volume translates into filling pressure, and is therefore the principal determinant of fluid tolerance. Impaired right ventricular systolic function, elevated right ventricular afterload, or right ventricular–pulmonary artery uncoupling all depress the cardiac function curve, producing disproportionate rises in RAP with small increments of preload [38]. Left ventricular diastolic dysfunction similarly causes filling pressures to rise steeply with small volume increments, leading to pulmonary congestion at modest fluid loads [39]. Heart rate modulates both: tachycardia shortens diastolic filling time and may limit the capacity to accommodate additional preload, while bradycardia extends it but reduces cardiac output at any given stroke volume. Because cardiac function also determines the ventricle’s position on the Frank–Starling curve, it is a shared determinant of both responsiveness and tolerance. Two patients with identical stressed volume may therefore differ markedly in tolerance depending on biventricular function and ventriculo-arterial coupling [37].

Second, venous capacitance and systemic compliance determine the relationship between intravascular volume and Pmsf. Sympathetic tone recruits unstressed volume to the stressed compartment, raising Pmsf without any change in total blood volume. Conversely, vasodilation (from sepsis, anaesthesia or vasodilator therapy) has the opposite effect. Capacitance is therefore not a fixed property but a dynamic modulator of venous return and, by extension, of congestion risk. Patients with identical fluid balance may exhibit different venous return profiles depending on prevailing vascular tone.

Third, transmission of venous pressure to organs reduces effective tissue perfusion pressure. Venous hypertension lowers renal perfusion pressure, raises interstitial pressure and may contribute to acute kidney injury despite apparently adequate systemic haemodynamics [40]. Elevated intra-abdominal pressure further turns fluid administration into a compartment problem, impairing renal, hepatic and respiratory function even when conventional haemodynamic variables appear acceptable [41].

Fourth, endothelial and glycocalyx injury, especially in sepsis, narrows the therapeutic window for fluid therapy. Increased permeability reduces intravascular retention of administered fluid, promoting interstitial oedema at lower volumes, while the fluid itself may contribute to further glycocalyx degradation, establishing a vicious cycle [42]. In Starling terms, net capillary filtration ordinarily rises with capillary hydrostatic pressure, but increased permeability shifts the filtration balance, allowing oedema to form even at normal hydrostatic pressure, thereby decoupling congestion from circulating volume.

Fluid accumulation syndrome is inherently a retrospective diagnosis [43]. Fluid tolerance, by contrast, is a real-time bedside assessment intended to identify early signals of cardiac-driven or volume-driven congestion before overt organ dysfunction develops.

### Bedside tools for assessing fluid tolerance

Recognising fluid intolerance requires integrating clinical examination, haemodynamic variables, imaging, and point-of-care ultrasound, with an emphasis on detecting rising downstream pressure and its relationship to cardiac function, rather than waiting for overt fluid accumulation syndrome.

CVP remains the most widely available surrogate for RAP. Its relevance lies not in predicting fluid responsiveness but in providing physiological context about downstream pressure within the venous return circuit, interpreted alongside cardiac output and signals of organ congestion [44]. A recent narrative review has argued for the continued clinical utility of CVP when interpreted alongside waveform analysis and trend monitoring [10]. Changes in the CVP waveform, particularly loss of the x-descent and an exaggerated y-descent, provide a bedside signal of right ventricular diastolic dysfunction [10]. In advanced decompensated heart failure, a CVP of at least 8 mmHg after intensive therapy, even with a preserved cardiac index, is strongly associated with worsening renal function [40].

Echocardiography sits at the centre of a monitoring strategy that integrates responsiveness and tolerance. Quantitative assessment of right ventricular systolic function (for example, tricuspid annular plane systolic excursion and fractional area change), right ventricular–pulmonary artery coupling, and left ventricular diastolic function grading (using E/A ratio, E/e′ ratio and left atrial volume index) provides the biventricular context needed to interpret both dynamic tests and markers of congestion [38, 39].

Right ventricular failure is a particularly instructive example: the failing right ventricle may remain preload-responsive on the ascending limb of its Frank–Starling curve yet generate disproportionate elevations in RAP with small volume increments, producing simultaneous responsiveness and intolerance [38]. In this setting, fluid administration may increase cardiac output modestly while accelerating systemic venous congestion. The clinical question is not whether stroke volume rises but whether net organ-level benefit is achieved.

The venous excess ultrasound (VExUS) score integrates inferior vena cava diameter with Doppler flow patterns of the hepatic, portal and intra-renal veins to grade systemic venous congestion from 0 to 3 [45]. Its principal advantage over CVP is that it provides organ-level information about the haemodynamic consequences of elevated venous pressure. Recent data have refined our understanding of what VExUS actually measures: high VExUS grades are closely linked to biventricular function rather than to volume status alone [46], and portal vein pulsatility integrates splanchnic capacitance, volume status, and biventricular function [47].

A conceptual reanalysis has argued that VExUS is therefore best interpreted as a marker of the interaction between cardiac function and venous return rather than as a pure index of fluid accumulation [37]. Similarly, its individual components are best considered alongside concurrent cardiac ultrasound assessment of biventricular function and ventriculo-arterial coupling, rather than in isolation [37, 48]. For instance, elevated VExUS scores have been reported in patients with cardiogenic shock despite near-normal fluid balance [36]. This distinction may help explain why a randomised trial of VExUS-guided decongestion in cardiorenal syndrome failed to improve kidney function recovery despite achieving reductions in cardiac-driven congestion [49], and illustrates the limits of a purely volume-centric interpretation.

The risk of fluid-induced congestion can also be estimated in some settings. In initially fluid-tolerant preload-responders, the portal vein pulsatility response to a fluid challenge, anticipated by PLR, identifies those who develop early systemic venous congestion [50]. More broadly, when fluid is administered, the trajectory of venous-congestion and lung-water signals tracks the cardiac-index response, with congestion developing far more often in fluid non-responders than in responders. Thus, responsiveness and tolerance, although dissociable, are physiologically linked [51].

Multi-region ultrasound may further refine assessment: lung ultrasound identifies pulmonary congestion; renal, portal, hepatic and inferior vena cava Doppler reveal early venous transmission; and common femoral vein Doppler offers a rapid superficial adjunct when abdominal windows are difficult [48, 52].

Pulmonary congestion is the most clinically apparent manifestation of left-sided fluid intolerance. Lung ultrasound B-lines provide a semi-quantitative, real-time estimate of extravascular lung water and correlate with pulmonary artery occlusion pressure [53]. More precise quantification is possible with transpulmonary thermodilution. Higher extravascular lung water indexed to predicted body weight is associated with worse outcomes across critically ill populations [54]. In acute respiratory distress syndrome, a maximum value above 21 ml.kg⁻¹ predicted 28-day mortality in one study [55]. In septic shock, extravascular lung water indexed to predicted body weight of at least 12.5 ml.kg⁻¹ within 24 to 48 h after initial resuscitation has also been associated with increased 28-day mortality [56]. In a general intensive care unit (ICU) cohort, higher maximum extravascular lung water independently predicted mortality [57]. Lung ultrasound and transpulmonary thermodilution therefore serve as markers of pulmonary fluid intolerance and, when abnormal, favour reassessment of the resuscitation strategy.

**Table 1:** Monitoring platforms for haemodynamic assessment of fluid responsiveness and fluid tolerance in critical care

Monitoring platform	Invasiveness	Contribution to fluid responsiveness	Contribution to fluid tolerance	Key limitations
Uncalibrated pulse contour analysis (e.g. FloTrac, ProAQT)	Minimal (arterial line)	Continuous SV; supports PPV, SVV and ΔSV from PLR, EEOT or tidal volume challenge	None directly	Inaccurate in arrhythmia, severe vasoplegia or with intra-aortic balloon pump; no congestion data
Calibrated pulse contour analysis with transpulmonary thermodilution (e.g. PiCCO, VolumeView)	Moderate (central venous and arterial thermistor catheter)	Continuous SV; supports all dynamic tests; reference CO via TPTD	Volumetric markers via TPTD: extravascular lung water, global end-diastolic volume, pulmonary vascular permeability index	Calibration drift between TPTD measurements; requires central and arterial access; tolerance signal intermittent
Pulmonary artery catheter (including continuous-CO variants)	High (right heart catheterisation via central vein)	Intermittent thermodilution CO (continuous via thermal filament in CCO variants); SvO2 as integrative marker of flow adequacy; PAOP a static, poor predictor of responsiveness	RAP, pulmonary artery pressures and PAOP provide information on right-sided loading, pulmonary hypertension and left-sided filling pressure	Highly invasive with non-trivial complication risk; intermittent CO unless continuous-CO variant used; PAOP confounded by airway pressure and ventricular interdependence; routine use not supported by outcome data
Focused echocardiography (TTE/TOE)	Non-invasive (TTE) or invasive (TOE)	LVOT VTI for SV estimation; ΔVTI with PLR or other dynamic tests	RV size and function, septal motion, LV diastolic function, IVC size and respiratory variation	Operator-dependent; intermittent rather than continuous; image quality limited in some patients
Point-of-care venous Doppler (VExUS, portal, femoral)	Non-invasive	None directly	Hepatic, portal and renal vein patterns; femoral vein flow morphology	Operator-dependent; thresholds derived primarily from cardiac populations; intermittent
Lung ultrasound (B-line scoring)	Non-invasive	None directly	Surrogate for extravascular lung water; tracks pulmonary congestion	Non-specific (positive in pneumonia, fibrosis and ARDS); intermittent
Central venous pressure with waveform analysis	Minimal (central line)	Static value a poor predictor of responsiveness; trends informative when integrated with flow data	Downstream venous return pressure; waveform abnormalities (loss of x-descent, exaggerated y-descent) signal RV dysfunction	Influenced by intrathoracic pressure and RV compliance; static value alone of limited value

CO, cardiac output; ΔSV, change in stroke volume; ΔVTI, change in velocity-time integral; CCO, continuous cardiac output; EEOT, end-expiratory occlusion test; IVC, inferior vena cava; LV, left ventricle; LVOT, left ventricular outflow tract; PA, pulmonary artery; PAOP, pulmonary artery occlusion pressure; PAP, pulmonary artery pressure; PLR, passive leg raising; PPV, pulse pressure variation; RAP, right atrial pressure; RV, right ventricle; SV, stroke volume; SvO₂, mixed venous oxygen saturation; SVV, stroke volume variation; TOE, transoesophageal echocardiography; TPTD, transpulmonary thermodilution; TTE, transthoracic echocardiography; VExUS, venous excess ultrasound score; VTI, velocity-time integral

### Application and interdependence of fluid responsiveness and fluid tolerance

Table 1 summarises the monitoring tools for fluid responsiveness and tolerance. Their interpretation remains conditional on measurement validity: PPV/SVV require appropriate ventilatory conditions; PLR and EEOT require real-time stroke volume or cardiac output tracking; and pulse-contour systems are influenced by vascular tone. When these conditions are met, and the signals are concordant, the available tools support probabilistic classification. None is accurate or precise enough to define responsiveness or tolerance from a single isolated value. In addition, the tolerance signals (VExUS, lung ultrasound and extravascular lung water) identify congestion or pressure transmission rather than absolute volume. Patients are therefore best placed within the phenotypes (Fig. 2) only when responsiveness, cardiac function, perfusion and congestion markers are concordant and reassessed serially.

At the bedside, these modalities are complementary rather than competing. In a patient with persisting shock after initial resuscitation, a pragmatic workflow begins with a dynamic test of preload responsiveness, selected according to patient conditions: PLR in most cases, or EEOT or tidal volume challenge in selected ventilated patients. This is followed by focused cardiac ultrasound assessment of biventricular systolic and diastolic function and ventriculo-arterial coupling. A systematic survey of congestion markers can then include the CVP waveform and trend, lung ultrasound for B-lines, and VExUS where abdominal windows permit. The decision to administer fluid is then conditioned on all three domains. A responsive, tolerant patient with low-to-moderate congestion markers and preserved biventricular function is the phenotype most likely to derive net benefit. Other configurations may favour vasopressor, inotropic or de-resuscitation strategies, depending on the broader clinical context.

This workflow is not an established standard of care; it is a pragmatic synthesis of guideline-supported dynamic responsiveness assessment, established critical-care echocardiography, and emerging congestion assessment. Integrating these into a single responsiveness/tolerance phenotype remains investigational, and its incremental value over usual care has not been tested in a randomised trial. Implementation is feasible where appropriate monitoring and trained point-of-care ultrasound are available. The principal barriers are operator dependence, intermittent sampling and uncertain congestion thresholds outside cardiac populations.

This integrated bedside approach to fluid status reflects a broader physiological point: fluid responsiveness and fluid tolerance cannot be assessed in isolation. Recent literature has established that fluid responsiveness and markers of venous congestion frequently coexist [7, 8]. A reanalysis of the VASST trial previously demonstrated that patients with septic shock and a CVP of 8 to 12 mmHg had higher mortality than those with a CVP below 8 mmHg [58]. In turn, a randomised trial of VExUS-guided decongestion in cardiorenal syndrome reduced cardiac-driven congestion without improving kidney-function recovery [49]. Together, these observations suggest two related points: first, clinically relevant venous congestion may emerge at lower thresholds than traditionally assumed; second, congestion markers reflect haemodynamic context rather than absolute fluid excess alone.

**Fig. 1:**
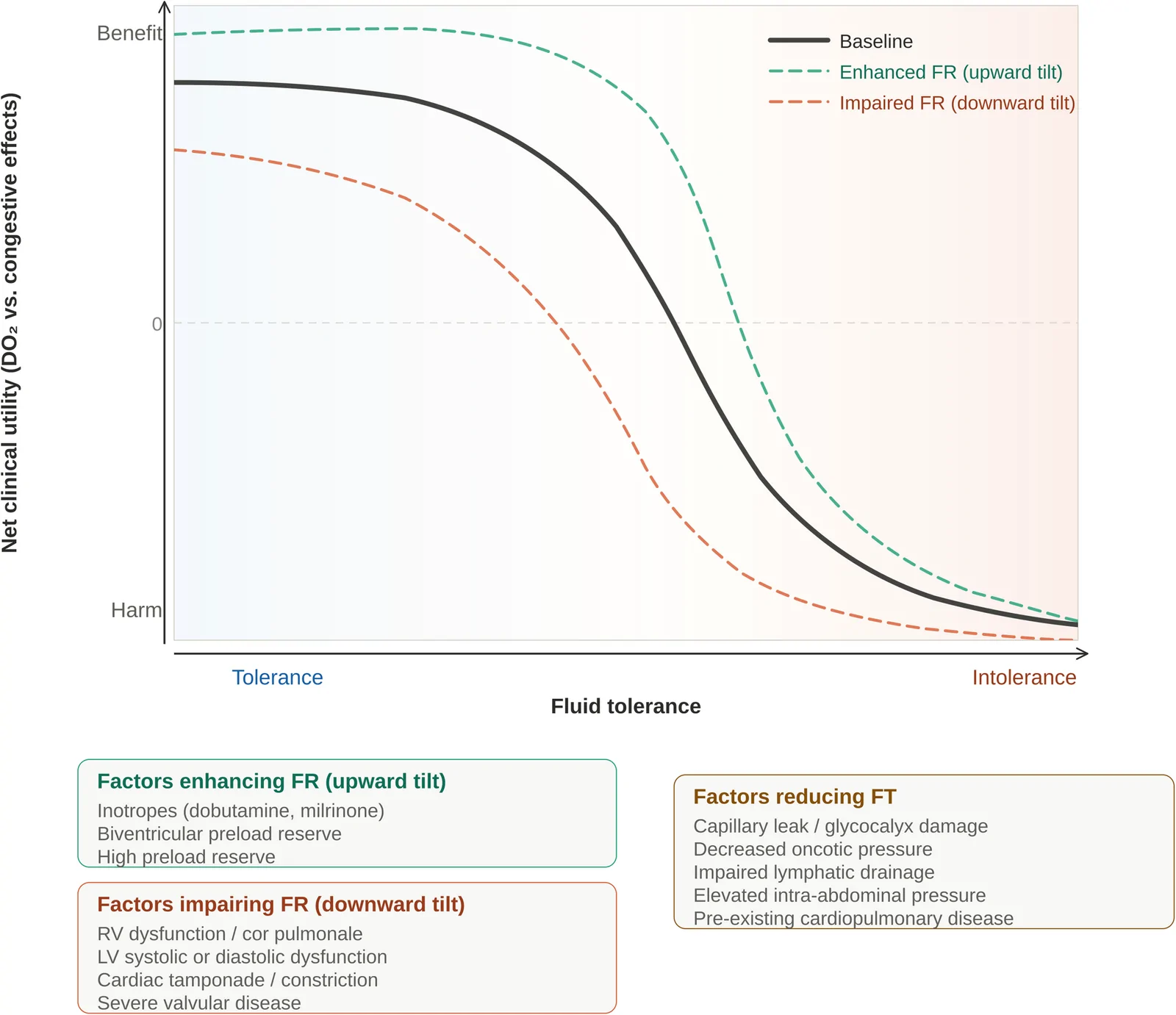
Conceptual relationship between the net clinical utility of fluid administration and fluid tolerance. This illustrative model depicts fluid management as a non-linear balance between the potential to increase oxygen delivery (DO₂) and the risk of congestive organ injury. Fluid responsiveness is primarily determined by position on the Frank–Starling curve and modulated by ventricular contractility, whereas fluid tolerance depends on biventricular function, venous capacitance, vascular integrity and venous outflow resistance. Because these domains share determinants, the two curves are physiologically interdependent rather than orthogonal; their crossover point marks the transition from net clinical benefit to net clinical harm and is expected to vary between patients and over time within the same patient

Rather than being truly independent, fluid responsiveness and fluid tolerance are best understood as dissociable but physiologically interdependent. They share determinants, principally biventricular function, stressed volume and venous capacitance, and therefore move in either parallel or opposite directions depending on which determinant dominates in a given patient. The conceptual balance between the potential to increase oxygen delivery and the risk of congestive organ injury may be represented as a non-linear interaction in which the net clinical benefit of fluid administration depends on where the patient sits along both the Frank–Starling curve and the cardiac-venous congestion relationship (Fig. 1).

**Fig. 2:**
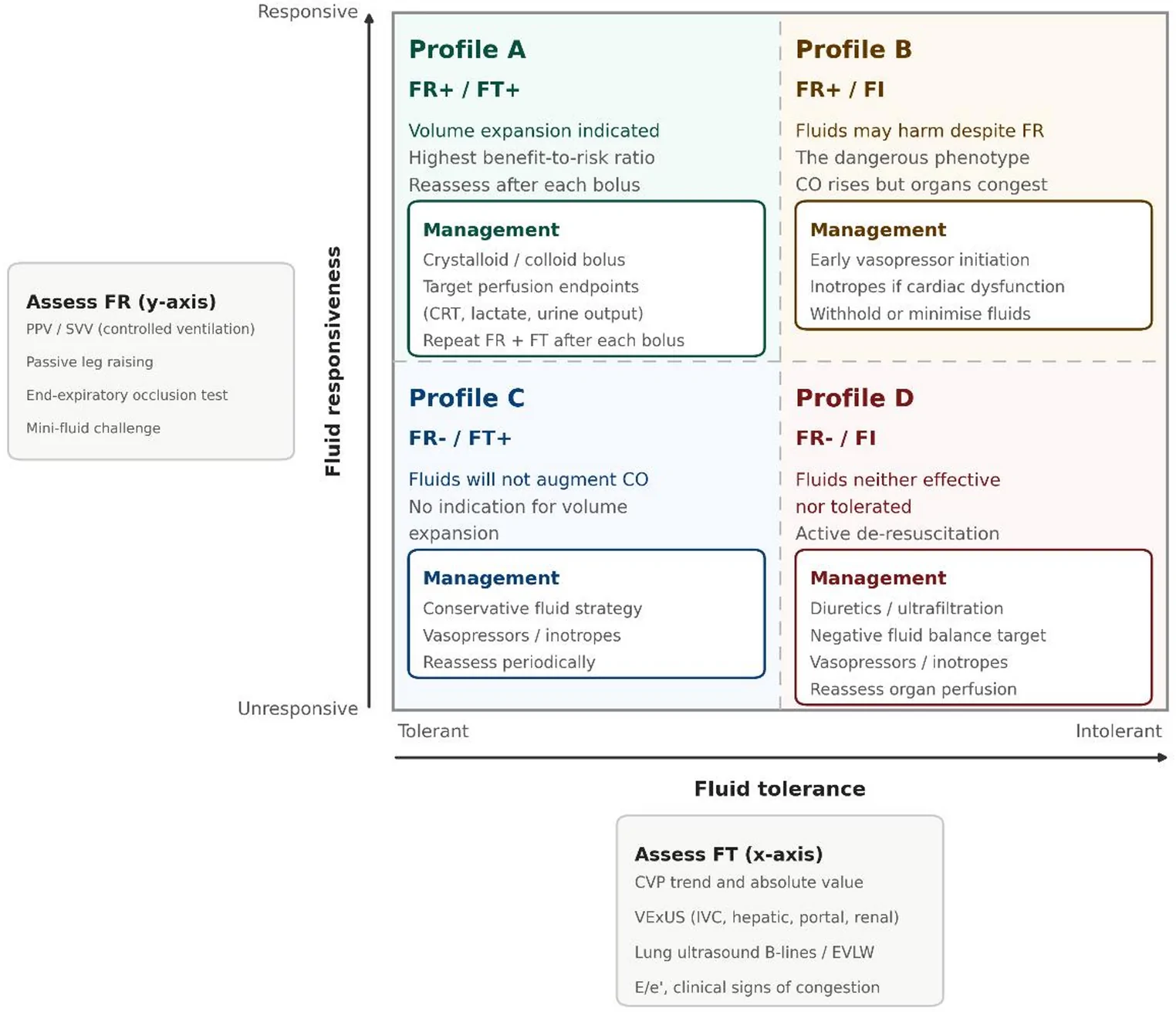
Heuristic framework for integrated assessment of fluid responsiveness and fluid tolerance. Four haemodynamic phenotypes emerge from joint assessment of fluid responsiveness (y-axis) and fluid tolerance (x-axis), each requiring a distinct management strategy. The axes are presented as orthogonal for didactic clarity; the two dimensions are in fact physiologically interdependent and share underlying determinants. Patients transition dynamically between phenotypes during illness, underscoring the need for serial reassessment. To avoid ambiguity in the abbreviations, the intolerant pole of the fluid-tolerance (x) axis is labelled fluid intolerant (FI) rather than FT-, and the fluid-responsiveness (y) axis runs from non-responsive to responsive

A patient with preserved cardiac function and moderate volume expansion may be fluid-responsive and fluid-tolerant; a patient with biventricular dysfunction may be fluid-intolerant even at low filling pressures, yet remain preload-responsive due to residual Frank–Starling reserve [7]. Conversely, a patient with substantial volume accumulation but preserved cardiac function may exhibit low congestion despite evident positive fluid balance [36, 37]. Combining the two dimensions at the bedside yields distinct haemodynamic phenotypes [59] (Fig. 2). Recent prospective data support this empirically: in a mixed ICU cohort of 138 patients, pulmonary and systemic congestion were dissociable (no statistical association) and independently associated with reduced left and right ventricular function, respectively, supporting in-parallel assessment of both signals [60]. 

At the tissue level, the dynamic interplay between fluid responsiveness and fluid tolerance is reflected in the variable perfusion effect of fluid administration. The recently proposed vascular waterfall framework offers a unifying conceptual lens, although its bedside operationalisation remains exploratory and requires prospective validation. Within this framework, flow depends not simply on the arterial-to-venous pressure gradient but also on whether arterial pressure remains sufficiently above a local critical closing pressure (Pcrit) to sustain tissue inflow (effective driving pressure: arterial pressure (Pa) − Pcrit) [61]. A fluid bolus may therefore improve macrocirculatory flow by increasing Pmsf, venous return, and cardiac output, yet tissue perfusion improves only if this also increases the effective microvascular driving pressure. Because local Pcrit, vasomotor tone and venous pressure transmission differ across organs, the same bolus may favour perfusion in one vascular bed while increasing congestion in another. Organs with limited buffering capacity, such as the kidney, are more directly exposed to venous hypertension [61, 62].

## Discussion

Reframing fluid therapy around the interaction between fluid responsiveness and fluid tolerance shifts the clinical focus from solely the forward-flow effect to the net physiological benefit, conditioned by the patient’s cardiac operating point. Accordingly, three priorities follow for bedside practice.

First, no single parameter or device adequately captures the complexity of fluid status. Dynamic indices of fluid responsiveness, quantitative measures of biventricular function, and markers of cardiac-driven and volume-driven congestion must be interpreted together. The choice of monitoring platform determines which of these signals can be observed simultaneously. Existing platforms differ widely in this respect (Table 1): some provide only flow variables, others include volumetric markers of congestion, and few currently integrate point-of-care ultrasound outputs. Technological advances that enable simultaneous visualisation of flow and congestion signals, supported by algorithmic pattern recognition, are likely to reshape practice over the coming decade.

Second, serial reassessment is essential. Patients transition between haemodynamic phenotypes as cardiac function, vascular tone and volume status evolve; a snapshot classification at ICU admission does not predict the patient’s status six hours later. A single PPV or VExUS reading is less informative than a trend over the resuscitation window, and protocols anchored to one snapshot risk under- or over-treatment.

Third, decisions are best conditioned on the patient’s prevailing cardiac operating point. Two patients with identical fluid balance and PPV may benefit from or be harmed by the same bolus, depending on biventricular function and venous capacitance. Matching the dynamic test to the patient’s physiology and matching the therapeutic response to the combined phenotype is a more defensible bedside standard than any fixed threshold.

A further qualification concerns the interpretation of dynamic responsiveness testing itself. A positive dynamic test, including PLR, identifies preload reserve under the prevailing conditions of ventilation, vascular tone and cardiac function. It does not by itself establish that oxygen delivery is inadequate, that oxygen consumption is supply-dependent, or that a rise in flow will outweigh haemodilution and capillary leak [63]. The markers used to infer the need for increased cardiac output or oxygen delivery are imperfect. Capillary refill time, lactate trajectory, urine output and central or mixed venous oxygen saturation are all confounded in sepsis by impaired extraction and microcirculatory shunting [64]. Thus, fluid responsiveness is best regarded as a necessary but not sufficient condition for fluid benefit [63]. When fluid is given on the basis of a positive test, the actual cardiac-output response and clinical effect should be reassessed rather than assumed [65]. The proportion of an administered bolus that remains intravascular, as opposed to being redistributed or extravasated, cannot be quantified directly at the bedside; in critical illness, capillary leak and endothelial permeability can increase fluid extravasation, promoting intravascular hypovolaemia and extravascular oedema [65, 66].

The commonly cited 10 to 15% PLR threshold is an operational convention derived from validation cohorts rather than a universal biological cut-off. Borderline values, measurement error, and patient heterogeneity materially affect classification, supporting the concept of a grey zone, in which results are verified serially and interpreted alongside the broader phenotype, rather than as a binary instruction to give or withhold fluid. Because preload responsiveness decays over minutes and shifts with sedation, vasopressor dose and ventilation, the manoeuvre is a single-point snapshot that must be repeated as conditions change [35]. The transient nature of PLR is intrinsic to the test rather than a flaw. The induced preload shift dissipates within minutes through venous stress relaxation and autoregulatory adjustment. This is why the signal must be captured in real time and confirmed to reverse; it is also what gives PLR its reversibility advantage over an irreversible bolus. Consistent with this, a key randomised trial of dynamic-measure-guided resuscitation reduced cumulative fluid balance, with secondary reductions in renal replacement therapy and mechanical ventilation, but was not powered for, and did not show, a mortality difference [67]. A meta-analysis of 18, mostly small, single-centre randomised trials performed for the 2026 Surviving Sepsis Campaign found a point estimate favouring lower mortality, but the confidence interval crossed no effect (risk ratio [RR] 0.91, 95% CI 0.79 to 1.06). It also found a statistically significant reduction in renal replacement therapy (RR 0.75, 95% CI 0.58 to 0.98). Therefore, the campaign’s recommendation for dynamic assessments was stated as conditional and based on low-certainty evidence [68]. These data support dynamic assessment as a reasonable stewardship strategy rather than as a proven survival intervention.

Most bedside tools, moreover, infer responsiveness from the cardiac-output response to a preload perturbation but do not directly quantify the determinants of venous return. Venous-return models that integrate mean systemic filling pressure, resistance to venous return and right atrial pressure, as originally proposed by Parkin and Leaning, are physiologically attractive because they describe volume state, vascular tone and cardiac function simultaneously [69]. Bedside estimation of mean systemic filling pressure is feasible but remains method-dependent and not yet routine [70], so these approaches are better regarded as an important research direction than as established bedside practice.

These priorities should be qualified by the limitations of the underlying evidence base. Much of the evidence for the interdependence of fluid responsiveness and fluid tolerance derives from single-centre observational cohorts, often in mixed medical-surgical populations and frequently enriched for cardiogenic shock. Randomised trials of monitoring-guided resuscitation strategies remain few, and no trial has yet tested a protocol that integrates responsiveness and tolerance into a single decision rule. Thresholds for the VExUS score, B-line counts and CVP derive predominantly from convenience cohorts. In a systematic review and meta-analysis, VExUS grades were associated with acute kidney injury and mortality in cardiac-surgical cohorts, but associations were weaker and inconsistent in general critically ill populations. This supports refining the score or interpreting its components before extrapolating beyond cardiac surgery [71]. Emerging emergency department data in suspected sepsis suggest that even mild VExUS abnormalities may identify patients at risk of later fluid accumulation syndrome, but these findings derive from a single-centre cohort and require confirmation in more severely ill shock populations [72]. Methodological heterogeneity in the definition of fluid tolerance limits pooling and transportability, and population-specific calibration of monitoring thresholds remains an open question.

Implementation also has workforce implications: quantitative echocardiography and VExUS scoring require dedicated training and credentialing, and protocols that depend on advanced point-of-care ultrasound may be difficult to deliver outside academic centres.

Future research should focus on defining monitoring-informed resuscitation targets. Priorities include validating composite fluid-tolerance scoring systems in non-cardiac intensive care populations, defining organ-specific congestion thresholds that predict harm, refining VExUS interpretation in the context of biventricular function, and testing whether algorithm-supported integration of fluid responsiveness and fluid tolerance into resuscitation decision-making improves patient-centred outcomes. The results of the multicentre Andromeda-VEXUS cohort nested within ANDROMEDA-SHOCK 2 may help clarify whether Doppler-identified venous congestion predicts renal replacement therapy or death in septic shock [73]. Such trials should be powered for realistic effect sizes and should report patient-important or organ-support outcomes, such as days alive and free of organ support. This is particularly important given the frequency of underpowered, neutral critical-care trials [74, 75]. The CONFIDENCE trial, for instance, randomises ventilated patients to lung-ultrasound-guided deresuscitation with a primary outcome of ventilator-free days and being alive at day 28 [76].

## Conclusion

Fluid responsiveness and fluid tolerance are best understood as distinct yet physiologically interdependent dimensions of the circulatory response to volume, with shared determinants. The framework in Fig. 2 presents them as orthogonal axes for didactic clarity; the four clinical phenotypes it depicts may be approximated at the bedside and may help structure personalised haemodynamic management [59]. Integrated haemodynamic assessment of critically ill patients may therefore be strengthened by combining dynamic tests of preload responsiveness with echocardiographic assessment of biventricular function and point-of-care markers of cardiac-driven and volume-driven congestion. As monitoring platforms mature, the most useful tools are likely to be those that integrate responsiveness and tolerance markers and support serial trend interpretation rather than snapshot evaluation. Whether monitoring-informed protocols built on this fluid stewardship framework improve patient-centred outcomes remains uncertain. Randomised trials of integrated monitoring protocols, including validation of fluid-tolerance scoring in broader critical care populations, are the necessary next step.

## Data Availability

No datasets were generated or analysed during the current study.
